# Aroma Characteristics of Green Huajiao in Sichuan and Chongqing Area Using Sensory Analysis Combined with GC-MS

**DOI:** 10.3390/foods13060836

**Published:** 2024-03-09

**Authors:** Lu Gao, Bolin Shi, Lei Zhao, Houyin Wang, Yake Xiang, Kui Zhong

**Affiliations:** Key Laboratory of Food Sensory Analysis for State Market Regulation, Agriculture and Food Standardization Sub-Institute, China National Institute of Standardization, Beijing 102200, China; gl18434764749@163.com (L.G.); shibl@cnis.ac.cn (B.S.); zhaolei@cnis.ac.cn (L.Z.); wanghy@cnis.ac.cn (H.W.); xiangyk@cnis.ac.cn (Y.X.)

**Keywords:** green huajiao, Sichuan and Chongqing area, aroma characteristics, sensory characteristics, partial least squares regression (PLSR)

## Abstract

Green huajiao has a unique flavor and is widely used in cooking as an edible spice. In this study, the intensity of overall aroma and aroma attributes of seven green huajiao samples from the Sichuan and Chongqing regions were evaluated using a dynamic dilution olfactometer and ranking descriptive analysis (RDA) technology. The volatile compounds and major aroma components were determined by GC-MS in combination with odor activity value (OAV) analysis. The partial least squares regression (PLSR) model was further used to identify the key aromas contributing to the aroma sensory attributes. Seven green huajiao samples were categorized into three groups: (1) huajiao samples from Liangshan have a strong intensity of pungent, floral and herbal aromas and a medium-high intensity of sweet aroma, and the key contributing aroma compounds were α-pinene, sabinene, β-pinene, myrcene, ocimene and linalool; (2) huajiao samples from Panzhihua and Hongya have a strong intensity of citrusy, lemony and minty aromas, and the key contributing aroma compound was linalool; and (3) the huajiao sample from the Chongqing region was categorized into a separate group and was characterized by a medium-high intensity of green, minty and sweet aromas, and the main aroma compounds are ocimene, citronellal and α-terpineol. These results provide useful basic data for evaluating the aroma quality and analyzing the key aroma characteristics of green huajiao in the Sichuan and Chongqing regions.

## 1. Introduction

Huajiao (*Zanthoxylum bungeanum* Maxim), the fruit of plants of the genus *Zanthoxylum*, a deciduous small tree or shrub, has been cultivated for more than 2000 years and is an endemic and resource-rich spice for traditional Chinese medicine in China [1,2,3,4]. According to the color of its pericarp, it can be divided into two main categories, red huajiao and green huajiao [5]. Green huajiao (*Zanthoxylum schinifolium* Sieb. et Zucc.), also known as fragrant pepper, cliff pepper, wild pepper, etc., has a strong and unique aroma that can impart an aromatic odor to food, and its dried ripe fruit rind has a unique hemp flavor, which is widely used in cooking as an edible spice [6,7]. Green huajiao is mainly cultivated in the Sichuan, Chongqing and Yunnan regions of China [8], and the varieties commonly used today are from Hongya, Jiangjin and Jinyang [9].

Aroma is the most important flavor characteristic of green huajiao and also a main index for quality evaluation, which determines its economic value. Huajiao samples are cultivated in about 20 provinces in China. Due to the influence of natural factors such as origin, soil and climatic conditions, the aroma of green pepper from different regions has different sensory and compositional characteristics, and the economic and application values also vary greatly.

Many reports have been published on the volatile compounds of huajiao and huajiao products. Different volatile compounds represent different sensory attributes. Gas chromatography–mass spectrometry (GC-MS) technology is widely used to identify and analyze volatile compounds in huajiao due to its high sensitivity and stability [10]. Not all volatile compounds contribute to the aroma of huajiao, and only some of volatile compounds can produce a particular aroma. These are called key aroma compounds and are determined by calculating the odor activity value (OAV) [11,12]. The OAV value combines the concentration of a volatile substance with an odor threshold value to enable a more objective evaluation. Volatile compounds with high OAVs are considered as potential contributors to sample flavor. Therefore, GC-MS technology in combination with OAV analysis is often used to qualitatively and quantitatively evaluate the role of aromatic compounds in dairy products, teas and spirits in terms of their contribution to the overall aroma [13,14].

In general, the result of aroma perception is mainly due to the overall effect of all important aroma compounds, so that the intensity of aroma perception cannot simply be reflected by the aroma concentrations of green huajiao [15,16]. Therefore, the aroma sensory analysis is the essential step in the evaluation of aroma characteristics. The aroma data from sensory analysis and GC-MS analysis complement each other, providing a more comprehensive and detailed analysis of the aroma characteristics of different origins. In combination with partial least squares regression (PLSR) or partial least squares discriminant analysis (PLS-DA) and other statistical methods [17,18], we can correlate the aroma compounds of the huajiao samples with its sensory attributes, and identify the key aroma compounds that contribute to the aroma sensory attributes.

In recent years, green huajiao has become very popular with consumers for its unique numbing aroma, and market demand is increasing rapidly [19]. However, the research reports on the aroma characteristics of huajiao mainly focus on red huajiao, and the research on the aroma characteristics of green pepper is relatively scarce; thus, it is necessary to investigate the aroma characteristics of green huajiao [17,20].

In this study, the aroma characteristics and sensory attributes of green pepper in the Sichuan and Chongqing regions were determined using GC-MS and sensory quantitative descriptive analysis. In addition, multivariate PLSR analysis was used to explore the correlation between the sensory attributes and aromatic active compounds of the characteristic aroma. This study will provide useful information for the identification of key aroma characteristics and sensory differentiation of various green pepper materials in the Sichuan and Chongqing areas, which could provide valuable information for regional distribution, quality grading and targeted application.

## 2. Materials and Methods

### 2.1. Samples and Chemicals

**Figure 1:**
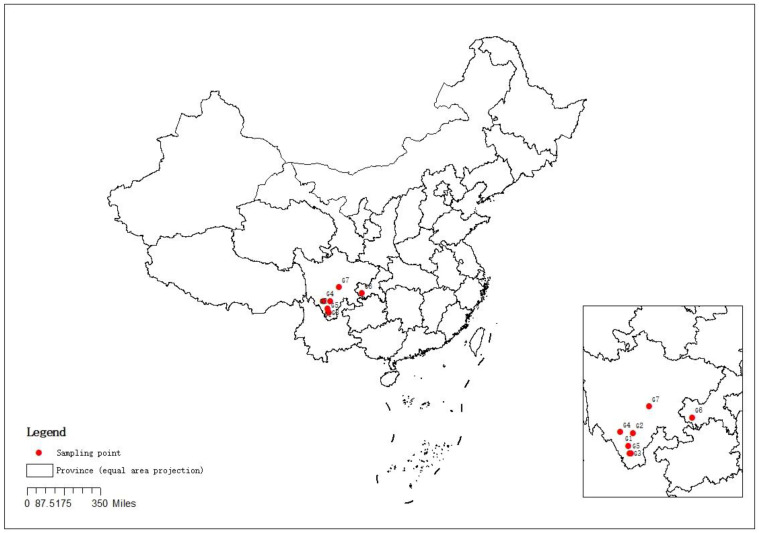
The geographical distribution of the seven green huajiao samples.

Seven samples of green dried huajiao grown in Sichuan Province and Chongqing City, China, and purchased at local farmers’ markets were used in this study. All samples were planted once a year and harvested around August 2022. The dried samples were sorted by hand to remove the closed huajiao, stems and impurities. They were then vacuum-sealed in a refrigerator at 4 °C and stored away from light. A sample of 10.00 g of dried huajiao was weighed and crushed (crush for 30 s, stop for 20 s, then crush again for 30 s), sieved through a 40-mesh sieve, placed in sealed bags and stored in a 4 °C refrigerator protected from light. All samples were crushed on the same day of use. The origin information for the 7 green huajiao samples is as follows: 6 green huajiao samples are from Sichuan Province, including 3 samples from Yousuo (G1), Lugu (G2) and Kara (G4) townships of Liangshan Autonomous Prefecture, 2 samples from Huangcao (G3) and Puwei (G5) townships of Panzhihua City, and one sample from Hongya (G7). The other sample is from Jiangjin (G6), Chongqing. The geographical origin and distribution of seven green huajiao samples are shown in Figure 1.

As an internal standard, 1-Nonanol (99.0%) was obtained from Shanghai Sigma Aldrich Trading Co, Ltd. (Shanghai, China), and the concentration was 1000 mg/mL. The N-alkane standards of the series (C4–C22) were purchased from Agilent Technologies Inc. (Palo Alto, CA, USA). In addition, lemon flavor, mint flavor, geraniol, cis-3-hexen-1-ol, vanilla flavor and cuminol were purchased from Sigma Chemical Co. (St. Louis, MO, USA).

### 2.2. Sensory Analysis

#### 2.2.1. Sensory Panel

The sensory evaluations of the green huajiao samples were carried out by a panel of eight trained assessors (4 males and 4 females, age between 24 to 28). The selection and training of eight trained assessors were according to the guidance of ISO 8586 [21] and ISO 5496 [22] standards. These assessors were familiar with the odors of the huajiao samples and underwent 10 h (4 sessions with a duration of 2.5 h each in a weekly cycle) of intensive training prior to the sensory evaluation of the huajiao samples. β-Pinene, menthol, terpinolene, linalool, limonene, linalool oxide, and myrcene were used as experimental samples to train the assessors. Only trained assessors who passed the assessment were selected to carry out the sensory evaluation of huajiao samples. All trained assessors signed a consent form before the sensory experiment, and this sensory study was reviewed and approved by the Ethics Committee of Tsinghua University.

#### 2.2.2. Overall Aroma Intensity Evaluation

The overall aroma intensity (OAI) was determined using a dynamic dilution olfactometer (AC’SCENT, St. Croix Sensory, Stillwater, MN, USA), which was used to definitively determine the odor thresholds of the samples using standard procedures and dynamic dilution of air samples in combination with measurements by a professional Olfactometer [23,24]. Accurately weighed 1.00 g of green huajiao particles were placed in a Tedlar@ gas sampling bag (volume 10 L, Cole-Parmer Instruments, Vernon Hills, IL, USA), the sampling bag was filled with clean air and stored at room temperature for 12 h for the determination. Eight trained aroma assessors took part in the evaluation. Linalool was used as a test sample to evaluate each assessor’s results until each assessor could accurately identify the odor, and the results were correct all three times. The gas flow rate of the olfactometer and the dilution ratio are shown in Supplementary Table S1.

#### 2.2.3. Aroma Sensory Attributes Intensity Evaluation

The aroma sensory evaluation environment complied with the requirements of the ISO 8589 standard [25]. The aroma descriptors of the green huajiao samples were defined according to the ISO 13299 standard [26]. Eight trained assessors generated eight aroma descriptors through several repeated discussions and tastings of green huajiao samples, and the reference literature was used to determine the aroma descriptors corresponding to the reference samples. The descriptor interpretations and reference samples are listed in Table 1. The aroma assessors were trained using the eight reference samples to fully know the eight aroma sensory attributes of the huajiao samples. Green huajiao particles (1.00 g) were weighed into a 250 ml brown bottle and left it at room temperature for 12 h for determination. Ranking descriptive analysis (RDA) is a fast descriptive analysis method that was developed on the basis of the flash profile (FP) method and has the advantages of fast analysis and simple data interpretation. Therefore, RDA was used to evaluate the sensory intensity of eight aroma attributes. Seven prepared huajiao samples were provided to the assessors at the same time. Each assessor had to rank seven huajiao samples according to the sensory intensity of the determined aroma attribute [27]. The evaluation was repeated three times for each assessor.

### 2.3. Isolation of Volatile Compounds by Solid-Phase Microextraction

Solid-phase microextraction (SPME) technology was used for the extraction of volatile compounds in green huajiao samples. An amount of 0.05 g of huajiao powder was accurately weighed into a 20 mL headspace vial, and 10 μL of internal standard solution (1-nonanol, 1.035 × 10^−2^ μg/mL) was added. The fiber (50/30 μm, PDMS/DVB/CAR; Agilent, Santa Clara, CA, USA) was inserted into the headspace of the sample vial and adsorbed at 65 °C for 20 min. After headspace extraction, the finished SPME injection needle was quickly inserted into the GC inlet and desorbed at 250 °C for 15 min. Each sample was repeated three times.

### 2.4. GC-MS Analysis

GC-MS analysis of green huajiao followed the method of a previous study [28]. The volatile compounds in the green huajiao samples were analyzed and identified by GC-MS (7890A-5975C, Mettler-Toledo Instruments Shanghai Co., Ltd., Shanghai, China). A chromatographic column HP-5MS (30 m × 0.25 mm × 0.25 μm, Agilent Technologies, Santa Clara, CA, USA) with a constant purge flow rate of 1.5 mL/min was used for chromatographic separation and identification of VOCs in the samples. The initial temperature of the column was 65 °C, ramped up to 90 °C at 25 °C/min and held for 1 min, ramped up to 105 °C at 1 °C/min and held for 2 min, ramped up to 200 °C at 3 °C/min and held for 2 min, and ramped up to 250 °C at 30 °C/min and held for 2 min. The carrier gas was helium, and the sample was injected using a split ratio of 20:1. The quadrupole MS conditions were: electron ionization source; electron energy 70 eV; MS detector temperature set at 230 °C; the temperature of the transmission line was 280 °C; the mass range was 35~500 amu. 

Qualitative and quantitative evaluation of volatile compounds was performed as follows. The volatile components were characterized by mass spectrometry, retention index (RI) was compared [29], and the MS results were searched using the NIST14.0 database, while compounds were characterized based on MS matches and structural information, and volatile compounds were identified if the matches were greater than 800. The actual RI values of the target compounds were compared to the standard RI values, and the standard RI values were matched to the NIST 14.0 mass spectrometry database. The peak times of the target compounds were matched with those of a series of n-alkanes (C4-C22) and the actual RI values were calculated using the following equation.

(1) $$RI=\left(n+\frac{t_{r}^{\prime }\left(x\right)-t_{r}^{\prime }\left(n\right)}{t_{r}^{\prime }\left(n+1\right)-t_{r}^{\prime }\left(n\right)}\right)\times 100$$

where *n* is the carbon number; *tr*′(*x*) is the retention time of the compound retained between carbon number *n* and *n* + 1 n-alkanes; *tr*′(*n*) is the retention time of n-alkanes with carbon number n; *tr*′(*n* + 1) is the retention time of n-alkanes with carbon number *n* + 1.

The results of the method with internal standards are relatively stable and the data are accurate. In this study, internal standards were added to semi-quantify the aromatic compounds in green huajiao. We used 1-Nonanol as an internal standard to calculate the relative concentrations of all volatile organic compounds, and the content of each compound was calculated using the following equation:

(2) $$Ci=\frac{Cis*Ai}{Ais*m}*Ws$$

where *C_i_* is the content of component *i*, mg/kg; C*_i_*_s_ is the concentration of the internal standard compound, μg/mL; *W_s_* is the mass of internal standard s, μg; *A_i_* and *A_is_* are the peak areas of component *i* and internal standard *s*, respectively; and *m* is the mass of sample to be measured, g.

### 2.5. OAV Analysis

The OAV is used to explain the contribution of a volatile substance to the overall flavor of a sample [28,30], which is calculated by the ratio of the concentration of the flavor substance to its odor threshold [31], and the calculation formula is as follows:

(3) $$OAV=\frac{C_{i}}{{OT}_{i}}$$

where *C_i_* is the concentration of a compound and *OT_i_* is the odor threshold of the compound (mg/kg). The thresholds used in this study were obtained by finding the threshold of perception of each volatile compound in water.

### 2.6. Statistical Analysis

The raw data from the experiment were statistically processed using one-way analysis of variance (ANOVA), the Mann–Whitney U test and the Duncan test using SPSS Statistics 25.0 software. Generalized Procrustes analysis (GPA) was used to analyze the sensory attributes of seven green huajiao samples. The correlation between the sensory attributes and the OAVs was analyzed using partial least squares regression (PLSR). Clustering heatmaps and plots were generated using Origin 8.0 (OriginLab, Northampton, MA, USA) and Chiplot online (https://www.chiplot.online). GPA and PLSR were conducted in XLSTAT 2019 (Addinsoft, New York, NY, USA). The experiment was repeated three times, and the mean standard error is used to represent all values; *p* < 0.05 was chosen as the level of statistical significance.

## 3. Results and Discussion

### 3.1. OAI Evaluation

**Figure 2:**
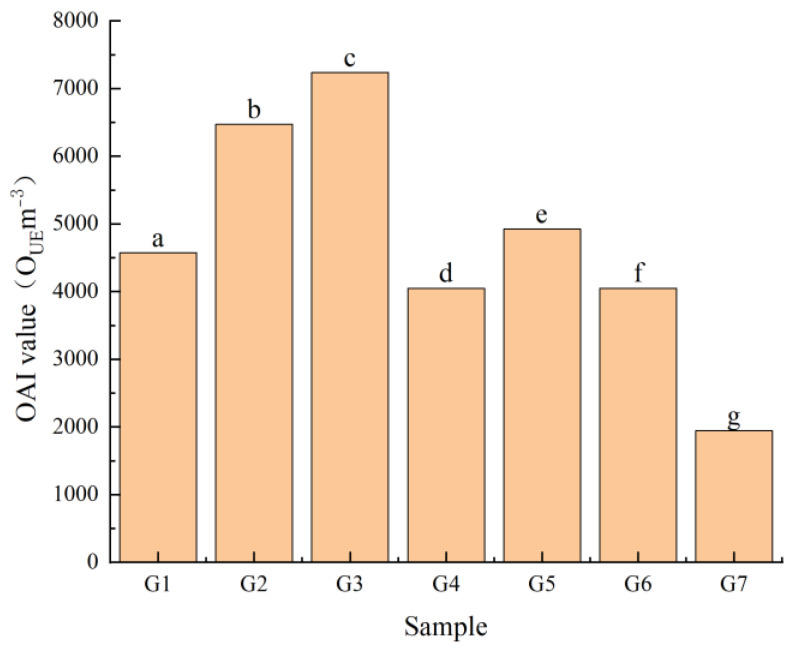
Histogram of overall aroma intensity of seven green huajiao samples. (Note: a–g indicates the significant difference in OAI values between the huajiao samples).

The OAI of the seven green huajiao samples was determined using a dynamic dilution olfactometer, and the mean value of the dilution ratio of each sensory assessor was calculated as the OAI value of the samples. As shown in Figure 2, the OAI values of the seven green huajiao samples showed significant differences (*p* < 0.05), ranging from 1944.4 O_UE_m^−3^ (G7) to 7237.3 O_UE_m^−3^ (G3). G2 had a high OAI value (approximately 6500 O_UE_m^−3^), and the remaining four samples were similar (approximately 4500 O_UE_m^−3^).

### 3.2. Aroma Sensory Attribute Intensity Evaluation

**Figure 3:**
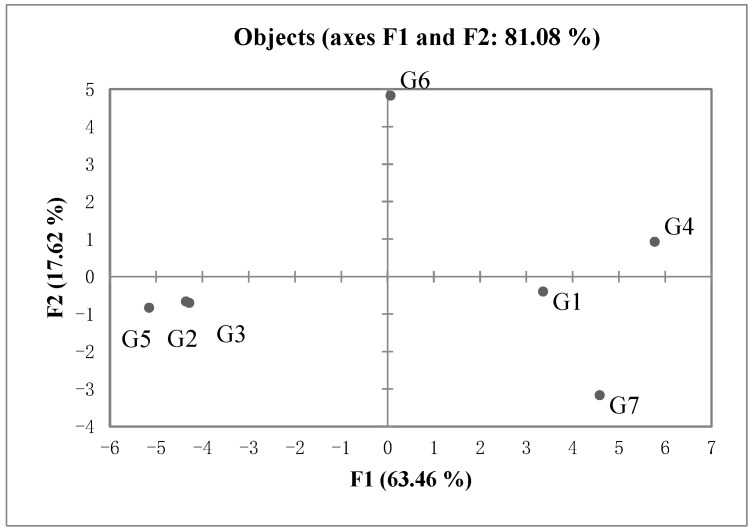
Score plot of green huajiao samples.

GPA was used to analyze and evaluate the aroma sensory attributes of green huajiao samples [32]. Figure 3 shows the score plots of seven green huajiao samples, and the first two principal components explained 63.5% (F1) and 17.6% (F2) of the total variance, respectively. Thus, the sum of the first two dimensions explained 81.1% of the total variance, indicating that the aroma characteristics of the samples can be expressed effectively. The seven samples are categorized into three groups: G1, G2 and G4, which are from the same area (Liangshan), are on the right side of the x-axis, indicating a good similarity among these huajiao samples; samples G3, G5 and G7 are very close to each other on the left side of the x-axis; and sample G6 from Chongqing City is located at the upper end of the y-axis and far away from the other green huajiao samples, indicating large differences in aroma attributes.

**Figure 4:**
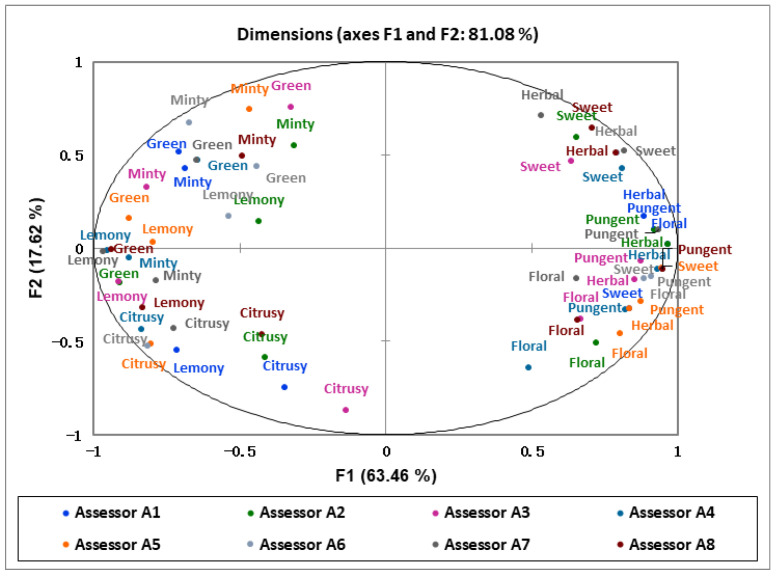
Load plot of sensory attributes of green huajiao samples.

A loading plot of aroma sensory attributes of the green huajiao samples is shown in Figure 4. Eight aroma attributes were mainly divided into two groups. Grassy, lemony, minty and citrusy are on the left side of the x-axis; and sweet, pungent, herbal and floral aromas are on the right side of the y-axis. Combined with Figure 4, G1, G2 and G4 showed a strong positive correlation with the attributes of pungent, floral and herbal aromas, while G3, G5 and G7 showed a strong positive correlation with the attributes of minty, lemony and citrusy aromas. In addition, G6 showed a higher positive correlation with the attributes of green, minty and sweet, and a weaker negative correlation with the attributes of citrusy and floral.

The rank-sum result of the RDA was then analyzed using the Mann-Kendall test and is shown in Table 2. There were significant differences (*p* < 0.05) in the aroma attributes of the different green huajiao samples. G1, G2 and G4 had strong intensities of pungent, floral, herbal and sweet aroma attributes, while G4 also had significantly higher intensity of herbs than G1 and G2. G3, G5 and G7 had strong intensities of citrus, lemon, green and mint attributes, whereby the intensity of green was significantly higher in G3 than in G5 and G7. G6 had a strong intensity of the green, minty and sweet aroma attributes and a medium-high intensity of the herbal attribute.

### 3.3. Volatile Compound Analysis

**Figure 5:**
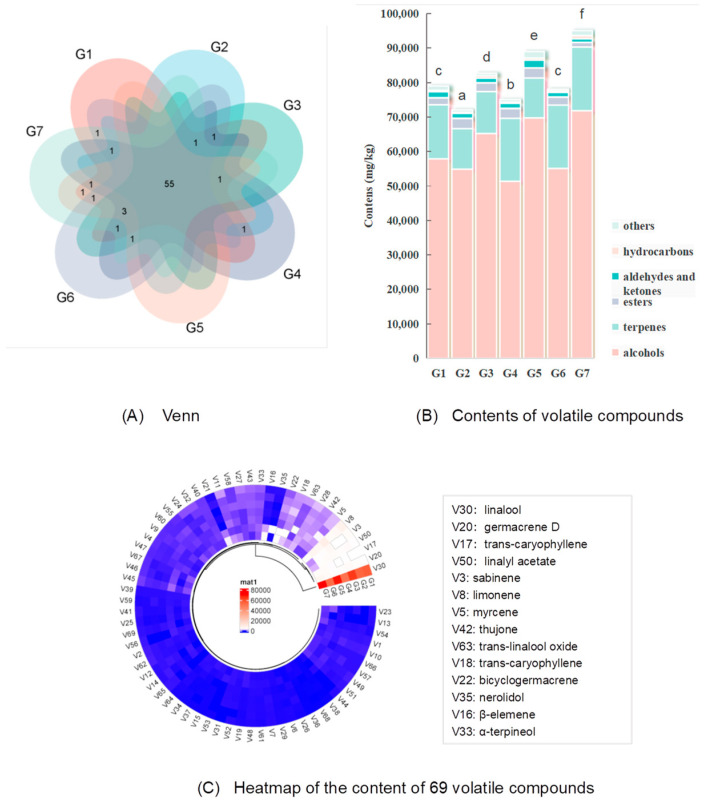
Overall characteristics of volatile compounds of seven green huajiao samples (**A**): Venn diagram; (**B**): contents of volatile compounds; (**C**): heatmap of the content of 69 volatile compounds). (Note: a–f indicates the significant difference in OAI values between the huajiao samples).

The volatile aroma compounds of seven green huajiao were separated and identified using HS-SPME and GC-MS, and the results are shown in Figure 5, Supplementary Figure S1 and Supplementary Table S2. A total of 69 volatile aroma compounds were detected in the seven green huajiao samples, including 55 common fractions (Figure 5A). Figure 5B shows significant differences in the content and composition of the volatile compounds in the seven green huajiao samples (*p* < 0.05), and the distributions of total volatile contents ranged from 74,006.16 mg/kg (G2) to 94,995.47 mg/kg (G7). G3 and G5 from the same city (Panzhihua, Sichuan Province) had higher volatile contents of 82,648.33 mg/kg and 88,871.57 mg/kg, respectively. The volatile substances of green huajiao were categorized into six groups, including 11 alcohols, 25 terpenes, 10 aldehydes and ketones, 7 esters, 7 hydrocarbons and 9 other substances. Alcohols and terpenes are the most important volatile compounds, and the percentage of these two compounds in huajiao samples is over 90%. Alcohols have the highest percentage content of 68.50% to 78.79%, followed by terpenes, ranging from 12.97% to 24.33%. The contents of aldehydes and ketones, esters and hydrocarbons were 1.15~2.52%, 1.41~4.15% and 0.48~0.91%, respectively. A previous report agreed with this result and showed that the content of alcohols and terpenes was higher in the volatile compounds of green huajiao, while the content of esters was higher in red huajiao [33]. Compared with red huajiao, the total volatile matter content of green huajiao from Sichuan and Chongqing regions was high, the proportion of alcohols was significantly high, and the proportion of esters was significantly low [17,18].

Figure 5C shows a heatmap of the content of 69 volatile compounds in the green huajiao samples. The volatile compounds with highest contents in the huajiao samples were linalool, germacrene D, trans-caryophyllene, linalyl acetate, sabinene, limonene and others. Other studies also reported that limonene, linalool and sabinene are the major volatile compounds in different varieties of huajiao and play an important role in the aroma characteristics [1,5]. In addition, a previous study reported that α-pinene and sabinene are the major volatile compounds in green huajiao [34]. It is assumed that these differences are due to the different cultivation areas, geographical and climatic conditions, varieties, processing and extraction technologies, etc.

### 3.4. OAV Analysis

According to Guadagni’s theory, aroma components in food products with a high aroma concentration and a low threshold value are probably the characteristic aroma of that food product. In general, the contribution of volatile compounds to the aroma characteristics of the samples in combination with the OAVs needs to be further determined [11,35,36]. The key volatile compounds were identified in each sample based on the OAV >1 [37]. A total of 27 key aroma substances were determined for all green huajiao samples, and the threshold values and aroma descriptions of these aroma compounds are shown in Supplementary Table S3.

As shown in Table 3, the OAV values of key aroma compounds in the seven green huajiao samples differed significantly (*p* < 0.05). The OAVs of three aroma compounds, myrcene(L4), limonene (L8) and linalool (L14), were the highest, exceeding 10,000. These three aroma compounds are reported as the main aromatic characteristics in the flavor of huajiao samples [4]. The OAV values of α-pinene (L1), sabinene (L2), 1,8-cineole (L9), ocimene (L10), linalool oxide (L12), α-terpinene (L13), thujone (L15), citronellal (L16), carvone (L21) and linalyl acetate (L22) were also higher in most huajiao samples, with OAVs between 1000 and 10,000. These results indicated that they are also the main aroma compounds contributing to the characterization of the aroma of most of the huajiao samples. The OAV of trans-nerolidol compound in sample G6 (Jiangjin, Chongqing) was significantly higher than that in the other Sichuan huajiao samples, which might be due to the different geographical locations.

**Figure 6:**
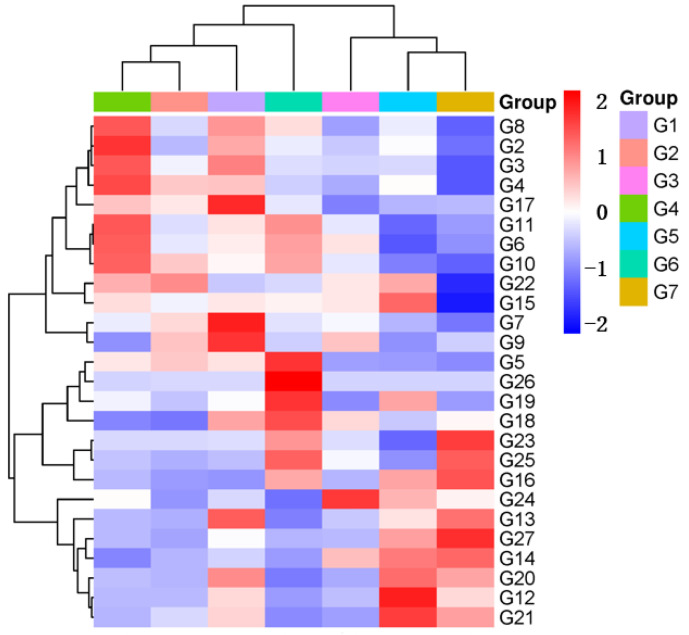
Clustering heatmap of OAVs of green huajiao Samples.

The clustering heatmap (OAV) of 27 key aroma compounds (OAV > 1) of seven green huajiao samples is shown in Figure 6, which illustrates aroma characteristic differences of varied huajiao samples. The color coding is graded according to the scale from blue (low OAV) to red (high OAV) [38]. The seven samples were categorized into three clusters. Cluster 1 included G1, G2 and G4. These three samples had higher OAVs of terpenes, including α-pinene (L1), sabinene (L2), myrcene (L4), limonene (L8), ocimene (L10) linalyl acetate (L22) and alcohols of 1,8-cineole (L9) and terpinen-4-ol (L17). These aroma compounds were the key aroma compounds that distinguished samples G1, G2 and G4 from the other samples [36]. Cluster 2 included G3, G5 and G7. These samples had higher OAV of linalool (L14), which is mainly sweet and floral. In addition, the compounds linalool oxide(L12), α-terpinene (L13), citronellal (L16) and carvone (L21) in samples G5 and G7 also had higher OAVs, while the compounds 1,8-cineole (L9) and α-terpineol (L18) in G3 had higher OAVs. G6 clustered by itself, with higher OAVs of phellandrene (L5), nerolidol (L26), trans-caryophyllene (L25) α-terpineol (L18) and carveol (L19).

Green huajiao from different sources exhibited differential main aroma compounds, which is more consistent with existing reports. Previous research has shown that limonene, cineole, sabinene and linalool were the main aroma components of green huajiao samples from Sichuan [39]. Other studies reported differentiated key aroma compounds in huajiao, which include limonene, linalyl acetate, β-pinene, hinokia and linalool [33,40]. It is said that the aroma compounds with higher OAV in huajiao oils from Hancheng, Shaanxi and Hanyuan, Sichuan, contain compounds such as β-laurenes, β-raisins, limonene and linalool, which can be used as key aroma compounds to distinguish different samples [37].

### 3.5. The Relationship between Aroma Attributes and Key Aroma Substances

**Figure 7:**
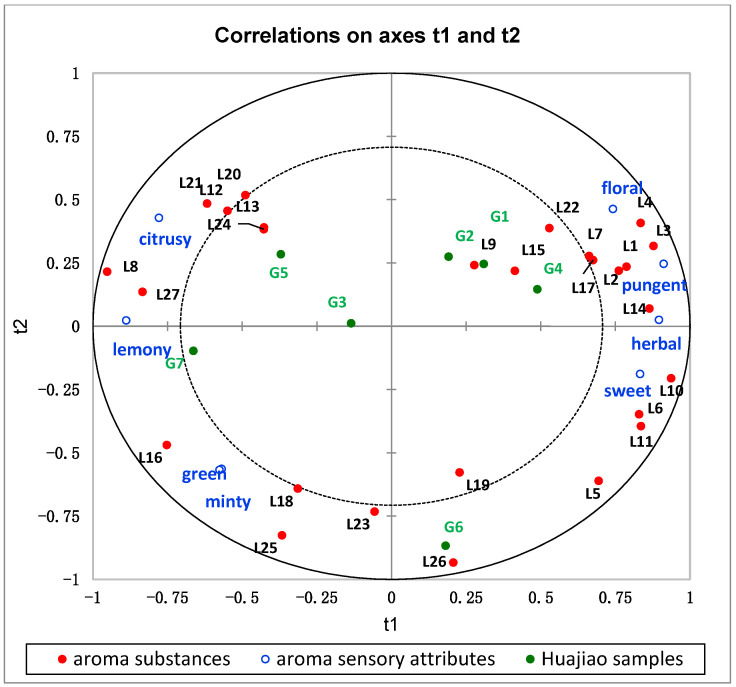
Bipolar diagram of key aroma compounds and aroma sensory attributes.

The correlations between 27 key aroma components (OAV > 1) and eight aroma sensory attributes of green huajiao samples were explained by the PLSR model [41]. The PLSR model was used to formulate a mathematical model to determine the relationship between the aroma attributes and the aroma components to identify the key aromas contributing to the aroma sensory attributes [37]. The OAVs of the key aroma components were used as independent variables and the rank sums of the aroma attribute as the dependent variables. As shown in Figure 7, the PLSR model presented two significant principal components that explained a total of 92.46% of the x variables.

Most of the key aroma components and aroma sensory attributes of the green huajiao samples were located between internal and external ellipses at r^2^ = 0.5 and r^2^ = 1, respectively, indicating that the model could explain these variables well, and most of the characteristic aroma components correlated differently with the aroma attributes of huajiao.

Eight aroma sensory attributes were distributed on the left and right side of the x-axis and showed a high correlation with the 27 aroma compounds. The sensory attributes floral, pungent and herbal are very close to each other and appeared on the upper right side of the coordinate axis, which showed a strong positive correlation with α-pinene (L1), sabinene (L2), β-pinene (L3), myrcene (L4), p-cymene (L7), linalool (L14) and terpinen-4-ol (L17). This is consistent with the previous report, which showed that L1, L2, L3 and L17 have pungent, herbal, pine oil odors and L4 has a flowery odor. The sweet sensory attributes are on the right side of the x-axis and show a strong positive correlation with the sweet odor of ocimene (L10). The sensory attributes minty and green are very close to each other at the bottom left of the coordinate axis. They showed a strong positive correlation with citronellal (L16) and a medium correlation with α-terpineol (L18), which is reported to have the odors of cucumber and mint, respectively. The sensory attributes citrusy and lemony were on the left side of the x-axis and showed a strong positive correlation with α-terpineol (L8), which is reported to have the odors of lemon and citrus. 

**Figure 8:**
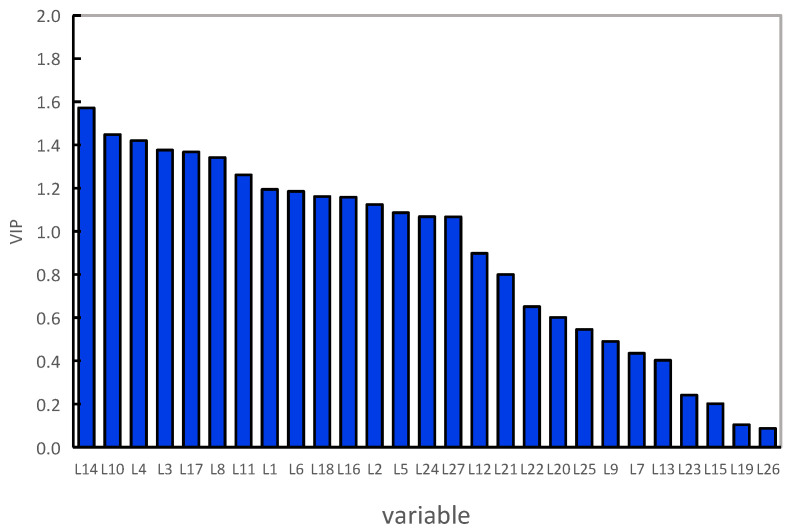
VIPs of the green huajiao samples.

The different aroma compounds of seven green huajiao samples were further analyzed using variable importance in projection (VIP). The VIP represented the contribution of the aroma compound to the differences between the huajiao samples, and substances with a VIP >1 were considered to make a key contribution to the differences between the samples [41]. As shown in Figure 8, 15 aroma compounds with VIP values greater than 1 (*p* < 0.05) were finally obtained, including α-pinene (L1), sabinene (L2), β-pinene (L3), myrcene (L4), phellandrene (L5), terpinolene (L6), limonene (L8), ocimene (L10), γ-terpinene (L11), linalool (L14), citronellal (L16), terpinen-4-ol (L17), α-terpineol (18), tetradecane (L24) and caryophyllene oxide (L27). Myrcene was identified as a key aroma compound to distinguish the cultivar and growing region of huajiao samples in a previous report [17]. In addition, linalool (L14), limonene (L8) and sabinene (L2) were also used to distinguish the growing area and cultivar [33,36]. These reports were consistent with the results of this study.

As shown in Figure 7, seven huajiao samples from Sichuan and Chongqing regions were divided into three groups. G6 from Chongqing was at the lower end of the y-axis had a medium-high aroma intensity of green, minty and sweet. Ocimene (L10), citronellal (16) and α-terpineol (18) were the key characteristic aroma compounds that distinguished the huajiao samples of Sichuan Province. Among the six huajiao samples from Sichuan, G1, G2 and G4, all from the same area (Liangshan), were located on the right side of the x-axis and were close to each other. They showed a higher intensity of floral, pungent and herbal and a medium-high intensity of sweet. α-pinene (L1), sabinene (L2), β-pinene (L3), myrcene (L4), ocimene (L10) and linalool (L14) were the key characteristic aroma compounds that differentiated these samples from other huajiao samples. In addition, G3 and G5 of Panzhihua and G7 of Hongya appeared on the right side of the x-axis and showed a high intensity of citrus and lemon aroma attributes. The key characteristic aroma compound was limonene (L8).

## 4. Conclusions

The aroma sensory attributes and volatile substances of seven green huajiao samples from the Sichuan and Chongqing regions were investigated in this study. In addition, the key aroma components that mainly contribute to the aroma sensory attributes of the different huajiao samples were studied. Compared to red huajiao, there are obvious differences in the aroma characteristics of green huajiao. The green samples from the Sichuan and Chongqing regions have different sensory properties and key flavor components. The aroma compounds of linalool, ocimene, myrcene, β-pinene, limonene and gamma-terpinene are the key aroma components to distinguish the samples from different areas of Sichuan and Chongqing. By studying the flavor characteristics of green huajiao in Sichuan and Chongqing, the differences in flavor of green huajiao samples from different origins were analyzed, and the correlation between green huajiao and physical and chemical substances was further explored. These results were useful and necessary for understanding the quality differences between different varieties of green huajiao in Sichuan and Chongqing and for characterizing the aroma traits between the varieties. The results of this study provide a theoretical foundation and basic aroma quality data on flavor quality for the development of the green huajiao industry and promote the local characteristic agricultural products industry and rural revitalization.

## Figures and Tables

**Table 1:** Aroma descriptors, definitions and reference samples for sensory evaluation.

Attributes	Definition	Reference Compounds
Citrusy	A compound aroma such as citrus, orange, grapefruit and other citrus fruits.	2 mL/L orange essence solution
Floral	A compound aroma of flowers, often with a sweet smell	1 mg/L geraniol alcohol-water solution
Green	A typical character of fresh fruit, vegetables or grasses, with an under-ripe odor	2.5 mL/L cis-3-hexen-1-ol solution
Herbal	A pungent odor similar to that of herbal plants	0.4 mg/L cuminol alcohol—aqueous solution
Lemony	Compound aroma of lemon-like fruit characteristics combined with acidity and gives a feeling of freshness and pleasant sensation	2 mL/L lemon essence solution
Minty	A stimulating odor with a cooling sensation, with a distinct felling of freshness	2 mL/L mint essence solution
Pungent	An irritating odor caused by spices such as anise, cinnamon, chilli and others	2 mL/L cinnamaldehyde solution
Sweet	A sweet and aromatic odor, similar to vanilla	2 mL/L vanilla essence solution

**Table 2:** Rank and GPA analysis of different green huajiao samples.

Attributes	Sum of Ranks						
	G1	G2	G3	G4	G5	G6	G7
Pungent	46	48	23	50	14	29	14
Citrusy	21	41	40	14	50	16	42
Floral	45	49	33	46	13	19	19
Lemony	20	17	45	22	48	25	47
Green	25	16	49	13	38	46	37
Minty	27	11	46	19	37	44	40
Herbal	40	43	14	52	21	38	16
Sweet	42	43	13	46	15	44	21

**Table 3:** OAV of key aroma substance components of green huajiao.

No.	Attributes	Sum of Ranks						
		G1	G2	G3	G4	G5	G6	G7
L1	α-pinene	5372	3398	2779	3474	2720	3164	1249
L2	sabinene	2719	1295	1426	3839	1934	1760	601
L3	β- pinene	963	647	570	1062	586	592	292
L4	myrcene	54,548	53,862	28,974	76,058	44,258	35,467	13,581
L5	phellandrene	551	592	325	539	314	870	286
L6	terpinolene	167	157	169	201	124	185	137
L7	p-cymene	694	406	333	320	229	306	134
L8	(+)-limonene	31,118	18,530	13,236	36,373	20,031	24,753	7904
L9	1,8-cineole	7858	5216	5216	2125	2125	3232	3232
L10	ocimene	2301	2645	2118	3364	1385	2874	1153
L11	γ-terpinene	83	72	74	108	51	98	60
L12	trans-linalool oxide	2036	890	975	910	4005	578	2016
L13	α-terpinene	3305	1320	1532	1398	2207	971	3122
L14	linathujonelool	51,508	49,364	58,776	46,232	63,237	47,766	64,740
L15	thujone	1618	1447	1605	1646	2156	1566	537
L16	(+)-citronellal	1431	1447	1493	1500	1797	1788	1948
L17	terpinen-4-ol	252	157	81	176	107	133	110
L18	α-terpineol	287	203	267	207	233	320	256
L19	carveol	29	25	21	28	35	43	22
L20	cuminaldehyde	127	67	64	70	136	50	118
L21	carvone	4851	2706	1276	1880	8684	728	6174
L22	linalyl acetate	1790	2826	2250	2625	2640	1884	782
L23	(-)-α-copaene	49	47	50	47	0	108	145
L24	tetradecane	136	105	243	155	188	90	159
L25	trans-caryophyllene	913	852	1119	943	753	1733	1757
L26	trans-nerolidol	156	126	31	82	32	6771	65
L27	caryophyllene oxide	23	9	12	12	38	11	56

## Data Availability

The original contributions presented in the study are included in the article, further inquiries can be directed to the corresponding author.

## Supplementary Materials

The following supporting information can be downloaded at: https://www.mdpi.com/article/10.3390/foods13060836/s1, Figure S1: Representative GC chromatograms of green huajiao sample; Table S1: Target gas flow and dilution ratio for dilution levels; Table S2: A single concentration of each major component in green huajiao; Table S3: Odor thresholds and aroma descriptions of key aroma substances.
