# Supplementary material for: Aroma Characteristics of Green Huajiao in Sichuan and Chongqing Area Using Sensory Analysis Combined with GC-MS
Source: Foods. 2024 Mar 9;13(6):836. doi: 10.3390/foods13060836 (PMC10969566; doi:10.3390/foods13060836)
Supplement: Supplementary file 1 [file foods-13-00836-s001.zip › foods-2895419-supplementary.pdf]

Supplementary Table S1. Target gas flow and dilution ratio for dilution levels

Dilution level	Odorous air flow（μL/min）	Carrier flow（L/min）	Dilution ratio
1	0.31	20.0	64000
2	0.63	20.0	32000
3	1.25	20.0	16000
4	2.50	20.0	8000
5	5.00	20.0	4000
6	10.0	20.0	2000
7	20.0	20.0	1000
8	40.0	20.0	500
9	80.0	20.0	250
10	160	20.0	125
11	320	20.0	63
12	630	20.0	32
13	1250	20.0	16
14	2500	20.0	8

Supplementary Table S2. A single concentration of each major component in green huajiao

No.	Name	Concentration (mg/kg)						
		G1	G2	G3	G4	G5	G6	G7
1	sabinene	2664.80±8.70	1269.05±5.51	1397.65±15.51	3762.00±112.98	1895.73±52.93	1724.87±3.13	588.53±42.63
2	(+) -limonene	6223.59±85.80	3705.97±332.95	2647.15±191.64	7274.59±218.95	4006.22±625.79	4950.58±74.56	1580.80±9.87
3	α-pinene	75.21±6.50	47.58±6.05	38.90±4.97	48.63±0.20	38.09±16.61	44.30±3.28	17.49±4.47
4	trans caryophyllene	1406.61±19.09	1312.46±70.78	1723.94±144.19	1452.75±119.19	1159.25±150.93	2668.28±191.16	2705.32±137.37
5	α-caryophyllene	631.71±1.73	584.88±36.37	907.68±82.43	600.47±40.98	545.32±64.25	1546.67±12.50	865.35±53.27
6	germacrened	1791.53±12.05	2375.11±153.92	3394.64±300.22	2342.28±156.39	1885.22±88.14	3172.84±36.90	4615.51±47.22
7	bicyclic macrobutylene	326.81±10.27	523.79±37.16	653.57±59.40	466.47±26.06	256.94±65.09	1287.28±61.42	1963.58±13.94
8	baichangene	293.44±17.12	70.76±7.43	100.79±16.34	72.21±2.20	127.24±14.27	123.02±13.15	230.02±36.68
9	phellandrene	22.03±1.27	23.69±12.91	13.02±0.97	21.57±7.77	12.57±4.99	34.81±4.50	11.44±4.41
10	terpinolene	33.50±5.68	31.38±8.75	33.80±0.79	40.26±11.48	24.90±5.98	37.06±1.53	27.31±1.60
11	ocimene	126.57±0.82	145.50±33.94	116.47±3.19	185.03±46.35	76.15±18.23	158.08±18.93	63.42±7.71
12	γ-terpinene	83.42±4.50	71.66±16.28	73.57±4.32	107.76±33.72	51.32±14.37	98.07±11.21	60.05±2.59
13	α-terpinene	661.00±6.06	264.05±21.32	306.34±29.04	279.60±2.80	441.39±8.12	194.24±6.33	624.47±15.37
14	linalool	55731.37±33.53	53411.70±508.20	63596.05±751.40	50022.73±366.69	68422.44±565.66	51682.42±896.97	70048.80±229.68
15	terpinen-4-ol	302.84±16.28	188.26±19.51	96.96±1.77	210.63±8.83	128.09±19.64	159.26±11.39	131.48±12.88
16	α-terpineol	344.05±16.55	243.81±26.91	320.04±14.38	248.73±9.37	279.73±14.76	384.05±3.62	307.45±1.72
17	myrcene	818.22±7.40	807.93±64.49	434.61±31.45	1140.87±47.89	663.87±0.69	532.01±1.99	203.72±19.33
18	thujone	582.40±21.36	520.93±16.68	577.82±40.85	592.51±17.11	776.27±7.64	563.61±33.32	193.27±30.06
19	ningketone	396.72±25.63	322.49±15.34	351.94±22.74	295.93±38.13	355.48±61.17	281.48±45.64	107.36±8.12
20	4-isopropylcyclohex-2-en-1-one	201.72±7.90	105.15±10.52	93.16±7.40	117.30±0.14	398.71±5.41	96.90±3.39	274.65±36.02
21	carvone	130.97±0.14	73.06±5.51	34.44±3.76	50.77±6.18	234.48±3.88	19.66±0.61	166.69±2.99
22	carveol	114.89±4.90	99.27±13.34	84.55±4.82	111.60±7.91	140.92±0.47	170.02±9.12	89.05±1.98
23	linalyl acetate	1790.40±77.14	2825.73±250.21	2250.37±153.58	2625.17±138.01	2639.51±315.35	1883.85±81.87	782.47±4.16
24	tetradecane	136.17±0.75	104.91±3.26	242.76±25.64	155.12±14.60	188.21±9.73	89.66±5.42	159.03±16.05
25	cetane	175.97±7.52	177.92±6.44	344.89±40.28	198.86±6.90	275.93±44.67	156.99±2.36	479.90±50.28
26	caryophyllene oxide	124.82±29.81	49.68±2.46	65.24±7.14	63.67±0.53	207.22±45.70	58.18±3.89	310.44±21.20
27	trans-linalool oxide	651.45±10.98	284.78±2.92	312.07±36.30	291.26±26.87	1281.63±71.69	184.93±8.92	645.03±20.03

Supplementary Table S3. Odor thresholds and aroma descriptions of key aroma substances

No.	Name	CAS	Chemical formula	Thresholds（mg/kg）	Aroma descriptions
L1	α-pinene	000080-56-8	C10H16	0.014	spice, herbal flavor
L2	sabinene	003387-41-5	C10H16	0.98	pepper, pine oil aroma
L3	β- pinene	000127-91-3	C10H16	0.14	pinewood, resinous incense
L4	myrcene	000123-35-3	C10H16	0.015	flowery
L5	phellandrene	000099-83-2	C10H16	0.04	Citrus aroma, mint flavor, black pepper aroma
L6	terpinolene	000586-62-9	C10H16	0.2	turpentine
L7	p-cymene	000099-87-6	C10H14	0.12	moist and moldy rag smell
L8	(+) -limonene	000138-86-3	C10H16	0.2	citrusy fragrance
L9	1,8-cineole	000470-82-6	C10H18O	0.064	turpentine, herbal flavor
L10	ocimene	013877-91-3	C10H16	0.055	sweet, vanilla fragrance
L11	γ-terpinene	000099-85-4	C10H16	1	turpentine, herbal flavor
L12	trans-linalool oxide	034995-77-2	C10H18O2	0.32	flowery
L13	α-terpinene	00099-86-5	C10H16	0.2	pine resin, mild woody fragrance
L14	linalool	000078-70-6	C10H18O	1.082	floral, green, woody and sweet aromas
L15	thujone	000546-80-5	C10H16O	0.36	mint fragrance
L16	(+)-citronellal	002385-77-5	C10H18O	0.025	cucumber fragrance
L17	terpinen-4-ol	000562-74-3	C12H16O3	1.2	huajiao, sweet and pine oil aroma
L18	α-terpineol	000098-55-5	C10H14O	1.2	citrusy fruit, mint flavor
L19	carveol	000099-48-9	C10H16O	4	retaining orchid flavor
L20	cuminaldehyde	000122-03-2	C10H14O	0.4	strong scent of kukui oil and grass
L21	carvone	000099-49-0	C12H20O2	0.027	retaining orchid flavor
L22	linalyl acetate	000115-95-7	C9H14	1	citrusy fruit aroma
L23	(-)-α-copaene	003856-25-5	C15H24	0.274	spice, herbal flavor
L24	tetradecane	000629-59-4	C14H30	1	——
L25	trans-caryophyllene	000087-44-5	C15H24	1.54	spicy, woody, citrusyaroma
L26	trans-nerolidol	040716-66-3	C15H26O	0.25	grass, flower and woody fragrance
L27	caryophyllene oxide	001139-30-6	C15H24O	5.5	——

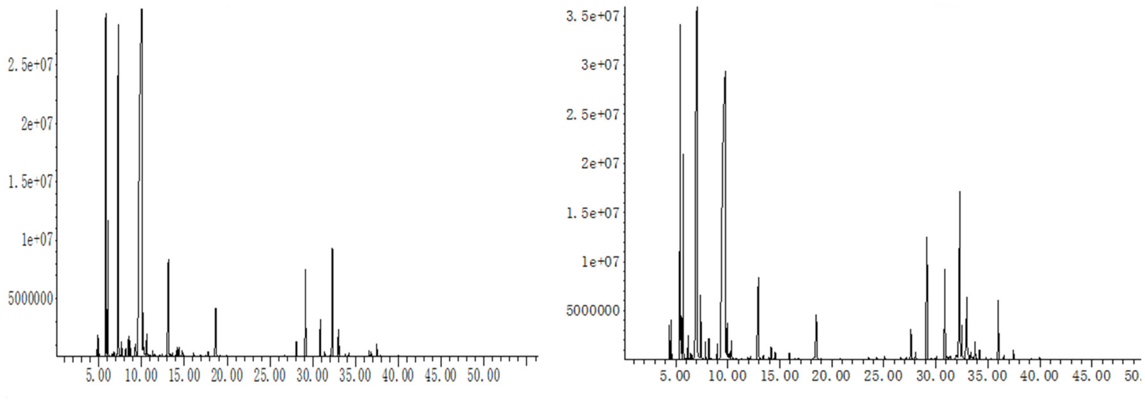

Supplementary Figure S1. Representative GC chromatograms of green huajiao sample.

(The GC diagrams of green huajiao from Miyipuwei green huajiao in Panzhihua City, Sichuan Province and green huajiao in Jiangjin District, Chongqing City, respectively)
